# The PRECISE (PREgnancy Care Integrating translational Science, Everywhere) Network’s first protocol: deep phenotyping in three sub-Saharan African countries

**DOI:** 10.1186/s12978-020-0872-9

**Published:** 2020-04-30

**Authors:** Peter von Dadelszen, Meriel Flint-O’Kane, Lucilla Poston, Rachel Craik, Donna Russell, Rachel M. Tribe, Umberto d’Alessandro, Anna Roca, Hawanatu Jah, Marleen Temmerman, Angela Koech Etyang, Esperança Sevene, Paulo Chin, Joy E. Lawn, Hannah Blencowe, Jane Sandall, Tatiana T. Salisbury, Benjamin Barratt, Andrew H. Shennan, Prestige Tatenda Makanga, Laura A. Magee, the PRECISE Network, Peter von Dadelszen, Laura Magee, Lucilla Poston, Sophie Moore, Rachel Tribe, Andrew Shennan, Tatiana Salisbury, Lucy Chappell, Sean Beevers, Ben Barratt, Meriel Flint O’Kane, Amber Strang, Marina Daniele, Kimberly Peven, Rachel Craik, Marleen Temmerman, Angela Koech Etyang, Sikolia Wanyonyi, Geoffrey Omuse, Patricia Okiro, Mary Amondi, Peris Musitia, Esperanca Sevene, Paulo Chin, Helena Boene, Corssino Tchavana, Eusebio Macete, Inocência Cuamba, Inácio Mandomando, Donna Russell, Joy Lawn, Hannah Blencowe, Veronique Filippi, Prestige Tatenda Makanga, Umberto D’Alessandro, Anna Roca, Melisa Martinez-Alvarez, Hawanatu Jah, Brahima Diallo, Ofordile Ogochukwu, Abdul Karim Sesay, Alison Noble, Aris Papageorghiou, Judith Cartwright, Guy Whitley, Sanjeev Krishna, Marianne Vidler, Joel Singer, Ehsan Karim, Beth Payne, Jing Larry Li, Jeffrey Bone, Domena Tu, Warancha Tumtaweetikul, Sumedha Sharma, William Stones, Alan Christoffels

**Affiliations:** 1 Department of Women and Children’s Health, School of Life Course Science, Faculty of Life Sciences and Medicine, King’s College London, 5th Floor, Becket House, 1 Lambeth Palace Road, London, SE1 7EU UK; 2 Nuffield Department of Women’s and Reproductive Health, University of Oxford, Oxford, UK; 3 Donna Russell Consulting, Seattle, WA USA; 4 Medical Research Council Unit (The Gambia) at the London School of Hygiene and Tropical Medicine, Fajara, The Gambia; 5 Centre of Excellence in Women and Child Health, East Africa, Aga Khan University in East Africa, Nairobi, Kenya; 6 Centro de Investigação em Saúde de Manhiça, Manhiça, Maputo Province, Mozambique; 7 Department of Physiological Science, Clinical - Pharmacology, Faculty of Medicine, Universidade Eduardo Mondlane, Maputo, Mozambique; 8 MARCH Centre, London School of Hygiene and Tropical Medicine, London, UK; 9 Department of Health Service and Population Research, Institute of Psychiatry, King’s College London, London, UK; 10 Lau China Institute, Faculty of Social Science and Public Policy, King’s College London, London, UK; 11 Department of Surveying and Geomatics, Midlands State University, Gweru, Zimbabwe

**Keywords:** Pregnancy, Africa south of the Sahara, Biorepository, Pre-eclampsia, Biological specimens

## Abstract

**Background:**

The PRECISE (PREgnancy Care Integrating translational Science, Everywhere) Network is a new and broadly-based group of research scientists and health advocates based in the UK, Africa and North America.

**Methods:**

This paper describes the protocol that underpins the clinical research activity of the Network, so that the investigators, and broader global health community, can have access to ‘deep phenotyping’ (social determinants of health, demographic and clinical parameters, placental biology and agnostic discovery biology) of women as they advance through pregnancy to the end of the puerperium, whether those pregnancies have normal outcomes or are complicated by one/more of the placental disorders of pregnancy (pregnancy hypertension, fetal growth restriction and stillbirth). Our clinical sites are in The Gambia (Farafenni), Kenya (Kilifi County), and Mozambique (Maputo Province). In each country, 50 non-pregnant women of reproductive age will be recruited each month for 1 year, to provide a final national sample size of 600; these women will provide culturally-, ethnically-, seasonally- and spatially-relevant control data with which to compare women with normal and complicated pregnancies. Between the three countries we will recruit ≈10,000 unselected pregnant women over 2 years. An estimated 1500 women will experience one/more placental complications over the same epoch. Importantly, as we will have accurate gestational age dating using the TraCer device, we will be able to discriminate between fetal growth restriction and preterm birth. Recruitment and follow-up will be primarily facility-based and will include women booking for antenatal care, subsequent visits in the third trimester, at time-of-disease, when relevant, during/immediately after birth and 6 weeks after birth.

**Conclusions:**

To accelerate progress towards the women’s and children’s health-relevant Sustainable Development Goals, we need to understand how a variety of social, chronic disease, biomarker and pregnancy-specific determinants health interact to result in either a resilient or a compromised pregnancy for either mother or fetus/newborn, or both. This protocol has been designed to create such a depth of understanding. We are seeking funding to maintain the cohort to better understand the implications of pregnancy complications for both maternal and child health.

## Background

The PRECISE Network is a new and broadly-based group of research scientists and health advocates mainly based in the UK and Africa. With core funding from the UK Research and Innovation (UKRI), we are establishing this network through a shared project investigating three important placental complications of pregnancy: high blood pressure (pregnancy hypertension), fetal growth restriction (FGR) and stillbirth. We estimate that about 46,000 women and two-and-a-half million babies (both before and after birth) die due to these problems every year, half of them in Africa [1]. In addition, about 50 million women and babies will have their short and long-term health altered because of these complications. These numbers represent one of the great global inequalities of our time.

**Fig. 1:**
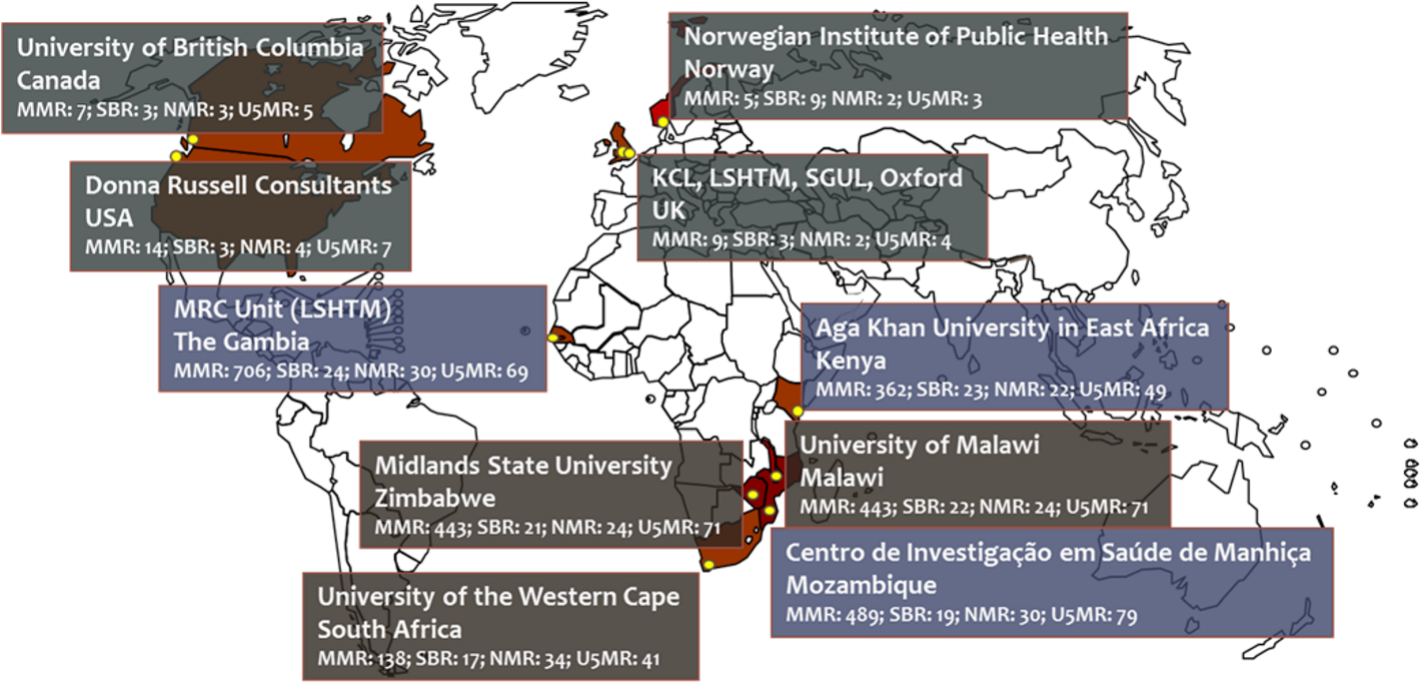
The geography of PRECISE. blue = active clinical sites; grey = methodology & co-ordination sites. *MMR*, maternal mortality ratio; *NMR*, neonatal mortality rate; *SBR*, stillbirth rate; *U5MR*, under-5 mortality rate

The co-investigator network has been established to deliver scientific excellence across the PRECISE objectives and broad programme of holistic, interdisciplinary pregnancy research. Figure 1 and the Network membership list (see Acknowledgements) show the global geography of institutions collaborating in this network. Our clinical activity is focused on West (The Gambia), East (Kenya) and South (Mozambique) sub-Saharan Africa.

This protocol paper is complemented by specific database and biorepository papers that provide detail related to data and sample collection methods, processing, monitoring and planned analyses.

## Aims and objectives of PRECISE

The broad objectives for The PRECISE Network cover all strategic areas of the programme: research capacity building, global maternal and child health research, partnership building and advocacy*.*

1. Build individual and institutional research capacity across Africa and the UK through a shared pregnancy research programme of work*.*
2. Develop a unique cohort of biologically and contextually characterised pregnant and non-pregnant women of reproductive age in East (Kenya), West (The Gambia) and Southern (Mozambique) sub Saharan Africa to support research into placental disorders (hypertension, fetal growth restriction and stillbirth) in the region.
3. Build sustainable, equitable partnerships across the individuals and institutions in The PRECISE Network, ensuring leadership and autonomy in research strategy and delivery across the collaborators.
4. Embed PRECISE in the global maternal and child health landscape across the areas of research, health service providers, NGOs, industry and national and international policy to maximise the contribution of PRECISE to the attainment of SDG 3 through broad advocacy and engagement*.*

The research outcomes planned within objective 2 (as above) are detailed as follows:

2.a To develop a unique cohort of pregnancies affected by placental disease and assess the prevalence of these disorders in women attending antenatal care in centres representative of urban and rural communities in three sub-Saharan African countries.
2.b To develop cohorts of women with unselected pregnancies and non-pregnant women of reproductive age, for comparison. These cohorts will provide appropriate data with which to compare the context of women and their biology as they have pregnancies complicated by placental disorders, or not. Sufficient culturally and geographically relevant data do not exist to identify pathways to pregnancy resilience or vulnerability, considering women’s burden of infectious and/or non-communicable disease. Existing control data have been almost uniformly from more-developed countries in Europe, North America and Australasia.
2.c To investigate environmental, biological, epidemiological, clinical, social/cultural and health system factors affecting the ability to understand, prevent and manage the effects of placental diseases for African women and their families.
2.d To investigate the potential for introduction of novel methods to assist the prevention, diagnosis and management of placental disorders in sub-Saharan Africa. Such methods could be new diagnostics based on the agnostic proteomic screening of samples from women with or without pregnancy complications. New pathways to disease may be identified that could be circumvented with novel therapeutics. In addition, the role of culturally and geographically relevant interventions (e.g. faith-based discussions around women’s autonomy of decision-making or the provision of roads and bridges to optimise access to health care) may be emphasised as critical health interventions.

## Methods

### Study design

This phase of the planned PRECISE initiative is a prospective observational cohort design. Where health issues are identified for individual women and newborns, then they will be referred into existing health system pathways. The funding mechanism for this cohort study specifically precluded clinical trials, enforcing the purely observational design. Once biomarker data become available for women after the clinical phase is completed, important clinical data (e.g., hyperglycaemia) will be provided to women and their caregivers. We are seeking further funding to begin intervention studies for pregnant and puerperal women, and their offspring, in these communities (e.g., stepped-wedge cluster randomised controlled trials).

### Research settings

**Fig. 2:**
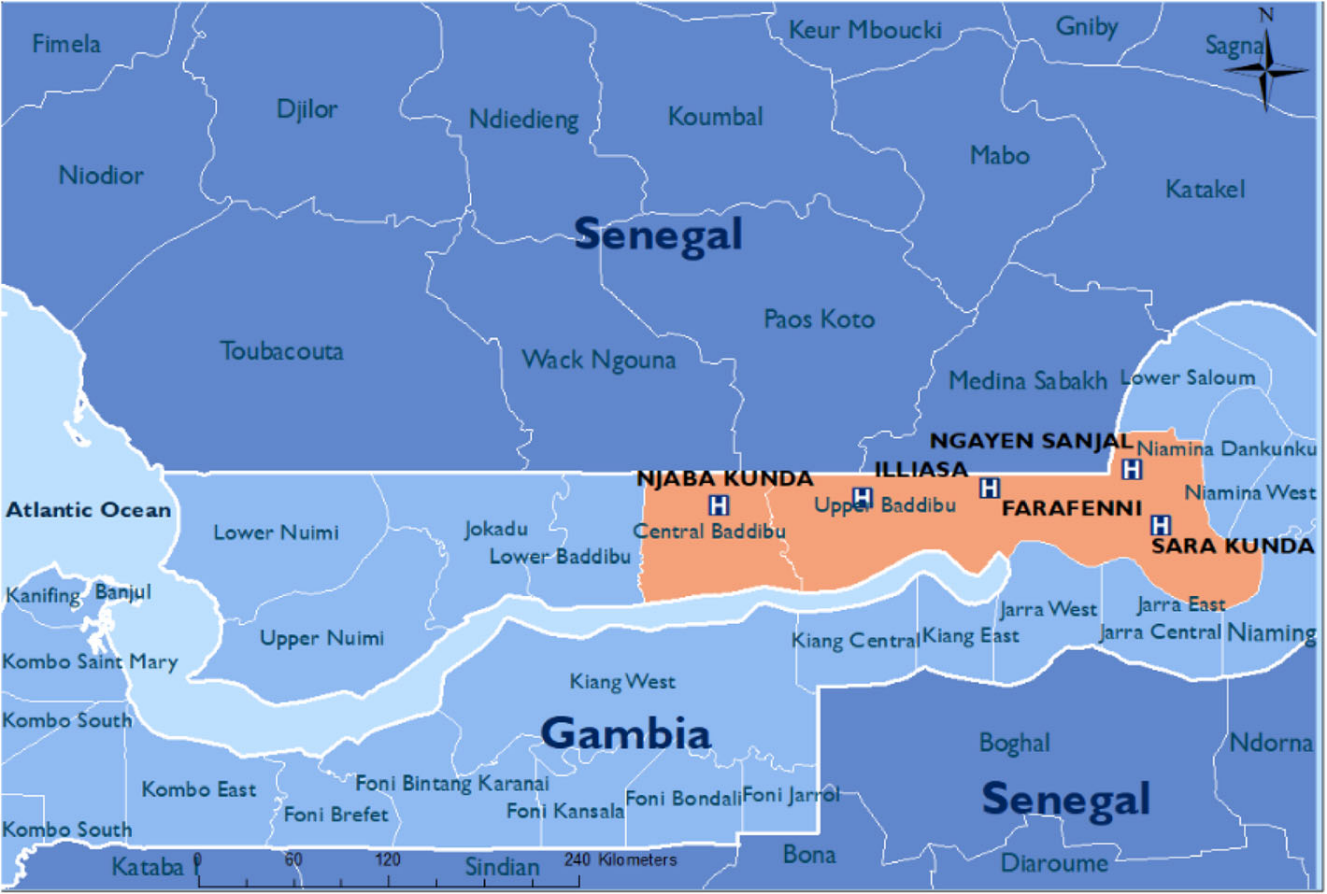
Gambian sites

**Fig. 3:**
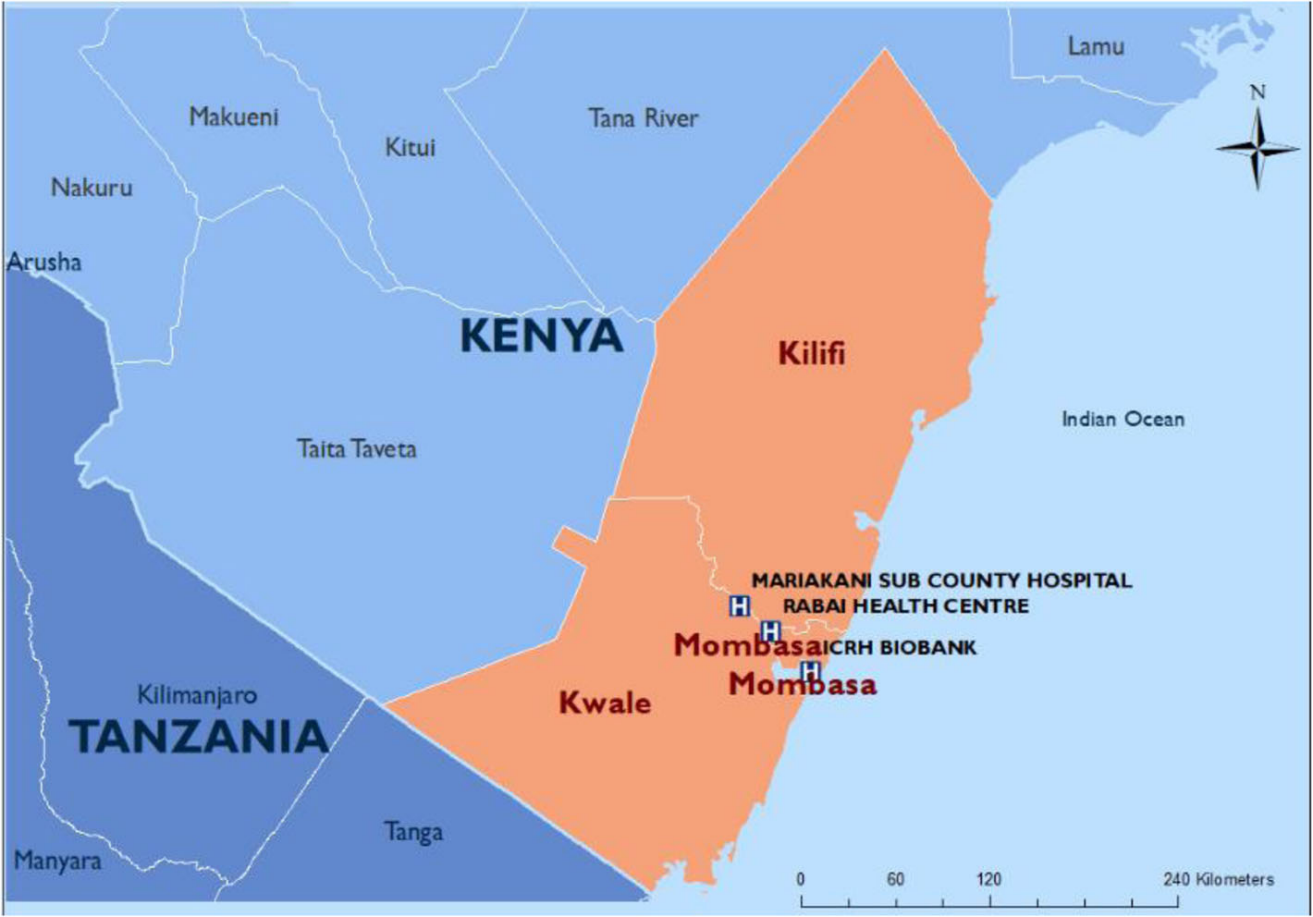
Kenyan sites

**Fig. 4:**
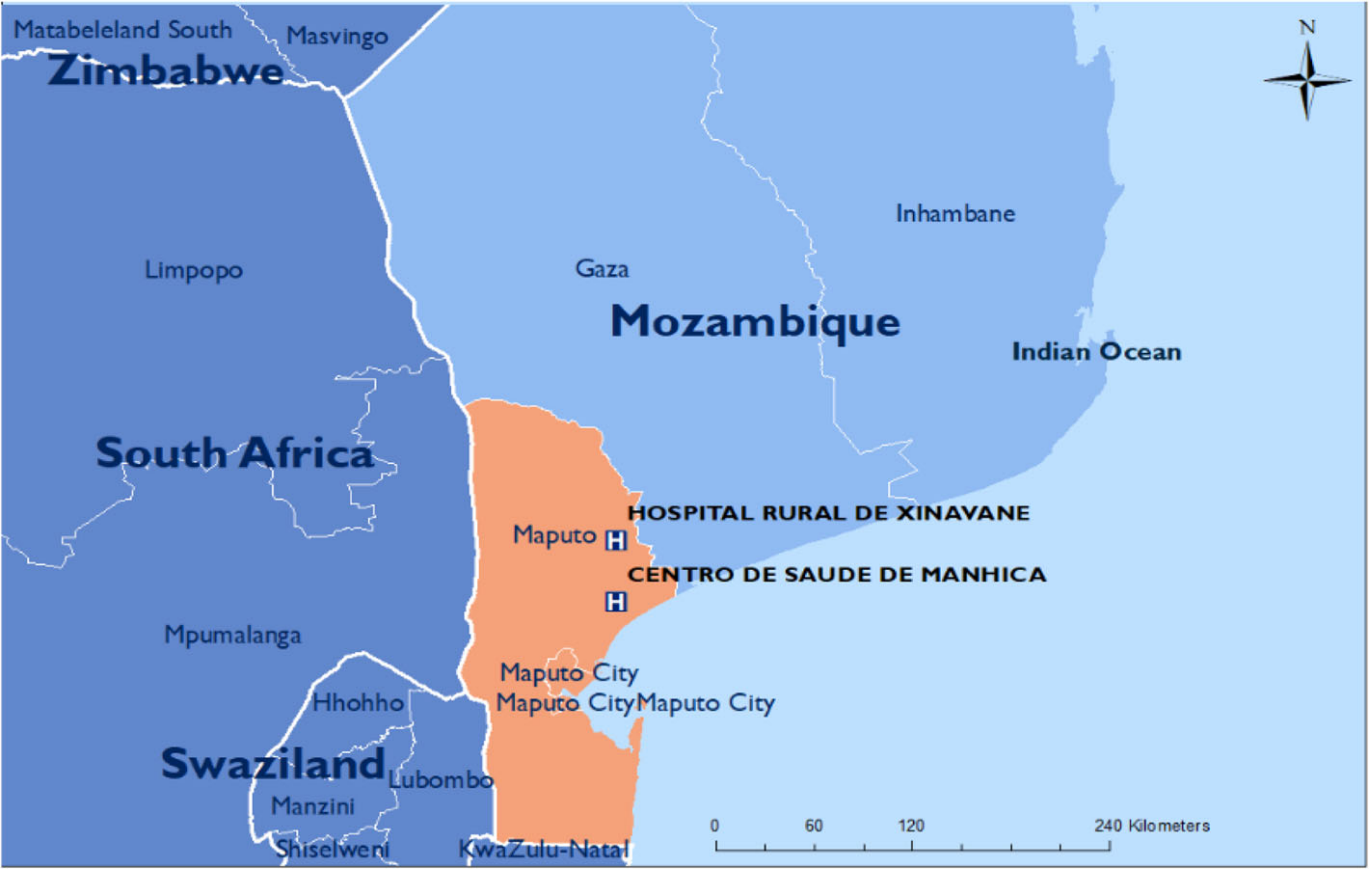
Mozambican sites

The PRECISE sites have been chosen in locations with research excellence and where data collection will produce representative cohorts of West, East and Southern African women. Given the transition towards increasingly urban populations in Africa and elsewhere in less-developed countries, PRECISE has been designed to recruit women living in both urban and rural settings in the three geographies (Figs. 2, 3 and 4).

*Specific variations on protocol implementation were made to respond to individual country scientific and ethical review boards’ recommendations.*

To maximise the quality of the biorepositories, each country’s selection of sites was undertaken to ensure rapid access to definitive biorepository facilities.

#### The Gambia (Fig. 2)

Our primary partner in The Gambia is the MRC Unit The Gambia at LSHTM. The field research will occur at the Farafenni Hospital (urban hospital) and associated rural primary health centres (PHCs) in Illiasa and Ngayen Sanjal; these health facilities cover a population under demographic surveillance since the 1980s.

#### Kenya (Fig. 3)

Our primary partner in Kenya is the Aga Khan University (East Africa) (AKU). The field research will be conducted through the Mombasa field station, with field activity in Mariakani Subcounty Hospital (urban) and Rabai Health Centre (rural).

#### Mozambique (Fig. 4)

Our primary partner in Mozambique is the Centro de Investigação de Saúde de Manhiça (CISM). The field research will occur in the Manhiça District Hospital (primarily urban population) and Xinavane Rural Hospital (primarily rural population), with some laboratory activity at Eduardo Mondlane University, Physiological Science and Pathology Departments in Maputo.

### The research participants

While PRECISE is designed to answer specific research questions, a major legacy will be the highly-phenotyped representative cohorts of West, East and Southern African women and the creation of an incomparable Africa-based biorepository for future hypothesis-generated and generating research. The core activity will be to collect environmental, social, geographical, demographic, clinical and biological data related to three populations of women.

Specific details of the planned cohorts are provided below.

### Research processes

#### Community engagement

The PRECISE Network clinical sites have been working in these communities for many years and have built strong relationships of trust with their communities. The local PRECISE leadership and study teams have informed participating communities of the study and, particularly, had in-depth discussions on the best approach within a given community for the collection of samples that may have cultural or religious significance such as maternal blood, cord blood and placental samples. In addition, the team has discussed with communities the best approach for collecting specimens immediately following childbirth (cord blood and placental tissue) and will return the placenta to the family (if that is their wish) after it is weighed and photographed, and small samples are taken from it.

Community engagement activities will be conducted in all study sites to ensure the women and communities in which they live are aware of the PRECISE programme of work. A brief overview of planned activity is presented in Table 1.

**Table 1:** Community engagement

	The Gambia	Mozambique	Kenya
Stakeholders to be engaged	Faith leaders Non-religious community leaders Women attending antenatal clinic (ANC)	Community leaders Pregnant women Women of reproductive age Mothers and mothers-in-law Partners Stakeholders (Ethical committees, investigators, policy makers) Nurses	Health care workers Community leaders Community health volunteers (CHV) Community-wide meetings Pregnant women, partners, family members
Methods of engagement	Go out into the community to engage with community leaders Open days at health facilities to explain to the women attending ANC	Health talks in the health facility Community meetings Informal conversations Discussion boards	Health talks (baraza) or videos on loop at ANC Community meetings (this will tag on to existing community-based meetings) Additional community meetings may be convened for target audiences (pregnant women, mothers, other in-laws, partners)
Key messages	Introduction to research Introduction to informed consent When/how/why storing biological samples	Assess the acceptability of the biorepository Promote early disclose of pregnancy and early attendance to the ANC visits. Promote birth preparedness and hospital delivery Identification of a health unit to give birth in Identification of skilled birth attendants	Introduction to research Introduction to informed consent When/how/why storing biological samples
Frequency of engagement	Sensitisation activities will occur before recruitment starts and continue during the recruitment period At the end of the study, we will communicate the results to the community following the same procedures	Health talks will be conducted twice a week during the morning with pregnant women in the health facility In the community meetings with specific groups will be conducted One meeting per month with the stakeholders	Initiate activities 2 months before data collection. Activities will be heightened in communities closest to the health facilities as they will have the largest number of women from these areas Activities will occur throughout the study. Each community will be approached roughly once per month

#### Consent process

Women enrolled in the PRECISE study will have an opportunity to read or be read the informed consent in their native language and to ask any questions they may have. Consent will be confirmed with the participant’s signature or a thumbprint. In the absence of a signature, a witness (other than the member of the research team obtaining consent) will be asked to sign. Finally, the member of the research team obtaining consent will sign the form. For recruitment of women aged 14 - 17 years old, the consent and assent processes will be guided by in-country ethics boards.

We will request tiered consent, whereby women will be asked to provide general consent, as well as specific vaginal swab consent, genetic study consent, the sharing of samples between African sites, the sharing of data and samples beyond Africa, and the sharing of data and samples with industry. It is specified that samples are only being taken for research purposes and will have no impact on their clinical care. The informed consent documents permit future research related to pregnancy and birth and their complications.

#### Vulnerable populations

This study involves pregnant women and newborns. In most sites, potential participants do not speak English. In these sites, consent will be taken in the local language to ensure that the potential participant understands the research. Every effort will be made to ensure their voluntary participation. It will be explained that participation is voluntary and can be terminated at any time without reason and without any penalty. If the potential participant has any questions, they will be answered in their native language to ensure that they understand the research and their potential role in it.

#### Confidentiality

To ensure privacy, consenting will take place in a private room at the local health facilities. Hard copies of any study-related forms, e.g. patient logs, will be stored in a locked cabinet in a storage room under supervision of the principal investigators (and destroyed after entering the database and performing the relevant quality control checks performed through the monthly data monitoring reports). Electronic records will be stored in password-protected computers and tablets and only approved study personnel will have access to entire level of information (e.g. laboratory technicians will have access only to a subset of data, thus reducing the risk for a major level breach). Access to the entire dataset will be restricted to the main PI and the site PIs, to keep a strict control of the data. All specimens and associated phenotypic data will be de-identified and given a unique participant identifier code and no personal information will be stored in the data management systems.

#### General points related to data collection

As described, the PRECISE research programme will collect 360° data that place women in their social, geographic, health services, nutritional and chronic disease contexts, as well as collecting important biophysical and biomarker data that will explain risks for, and pathways to, placental disorders and other complications of pregnancy. Details of the data fields being collected are in the complementary database paper [2].

Using mixed methods, we will assess women in terms of their beliefs and practices around pregnancy, access to care and the acceptability of novel diagnostic tools and interventions. Similarly, we will assess these factors in men’s groups, mothers and mothers-in-law, faith and community leaders, and health service providers and administrators. Much of this work has already been completed in Mozambique during the CLIP trial feasibility study [3–6]. This activity will be supplemented in Mozambique and replicated in The Gambia and Kenya. Additional details are provided in the qualitative methods paper [7].

Non-clinical data will relate to their nutritional status, demographics and women’s natural and built environment, this includes data on the women’s home including the structure, building materials, proximity to a toilet as well as the environment including cooking facilities and water availability. In addition, we will capture data on available health services (and an assessment of the quality of care provided by them). All data will be entered into the PRECISE data platform. These data will largely be collected during the enrolment visit for all women, whether non-pregnant or pregnant.

Clinical data will be collected using an electronic data collection platform with the additional PRECISE data fields. At enrolment, these data will include limited information about past obstetric, medical, and surgical history for all women. For pregnant women, best estimates of pregnancy dating will be obtained at the first visit. At all visits, women will have their weight, blood pressure (BP) and SpO_2_ measured. Full details on the data being captured are outlined in the database paper [2].

Specific clinical tools to be tested and validated within PRECISE include: (i) the TraCer (transcerebellar diameter) app that will date all pregnancies using the highly-conserved and growth restriction-resistant transcerebellar diameter (separate in-country, sub-project-specific ethics approval for the development and utility testing of the app has been granted) (pregnancy only); (ii) the CRADLE VSA semi-automated and validated BP device will be used for all clinical measurements of blood pressure (BP) in the study [8–14] (non-pregnancy and pregnancy); (iii) pulse oximetry will be used to assess the impact of cardiorespiratory disease [15–21] (non-pregnancy, pregnancy and neonates); (iv) the PIERS On the Move (POM) platform to provide time-of-disease risk estimates to hypertensive pregnant women using the PIERS (Pre-eclampsia Integrated Estimate of RiSk) models [15, 22–35] (non-pregnancy and pregnancy); and, possibly, (v) WOICE, the WHO Maternal Morbidity Working Group Tool [36–39] at visits in the third trimester.

Standards for the use of the CRADLE BP device, pulse oximetry and TraCer platform will be detailed in the relevant portions of the clinical SOP.

### General points about consent

In all countries, women aged 18 years old or older can provide informed consent for research, and pregnant women aged 16 and 17 years old can provide informed consent. In Mozambique, pregnant women aged 14 and 15 years old can provide assent for research with consent provided by a custodial adult. The Department of Health in Kilifi Country has asked us to replicate this in their jurisdiction, and the required ethical amendments are in progress. For all cohorts, we have set an upper age limit at 49 years of age. We will exclude any women who have already been approached and declined participation in PRECISE.

#### Cohort 1: non-pregnant women of reproductive age (Fig. 5)

In each country, 50 women will be recruited each month for 1 year, to provide a total national sample size of 600 non-pregnant women of reproductive age. These women will provide culturally, ethnically and spatially-relevant control data with which to compare women with normal and complicated pregnancies (PRECISE Objective 2b). Recruitment will occur throughout the year to provide seasonal data, with the associated fluctuations in geographical (due to seasonal migration) and infectious disease burdens of risk.

**Fig. 5:**
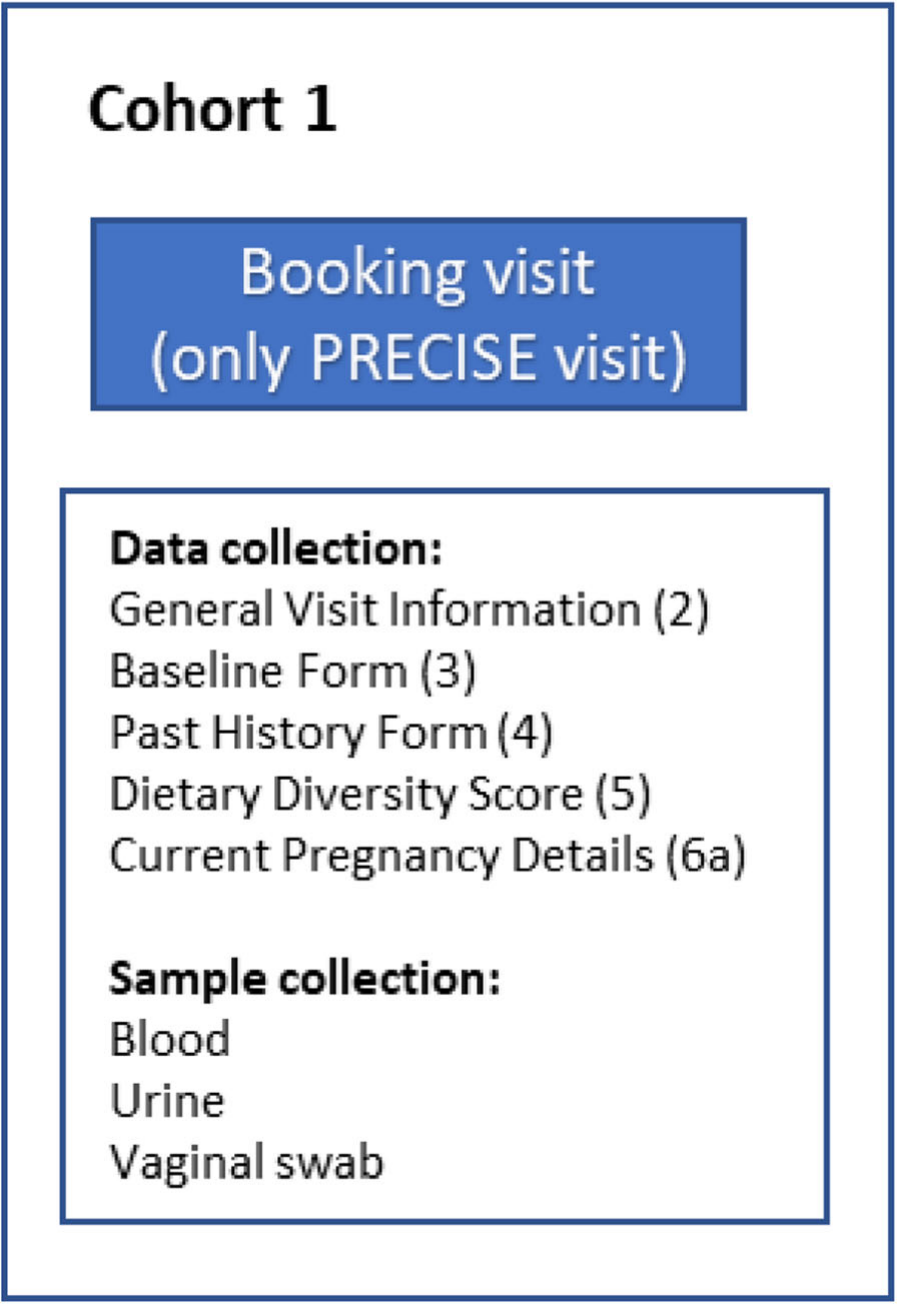
Flow and sampling of nonpregnant women of reproductive age

#### Cohort 1: inclusion criteria

- Attending facility for the care of someone else (e.g. infant vaccinations) or their own care (family planning assessment) or in the community (using the DHS infrastructure).
- Not pregnant by self-report (confirmed by a pregnancy test) or within the last 6 months by self-report (including miscarriage, termination, stillbirth or live birth).

These women will be approached either in the community (in The Gambia) or when attending the facility for the care of someone else (e.g. for infant vaccinations) or for their own care (family planning assessment) (Mozambique and Kenya), and asked to provide data and samples at a single time point. Non-clinical and clinical data as well as biological samples will be collected during the first year of the clinical phase of the project.

At the encounter, consenting women will be asked to provide:

- Demographic, non-clinical and clinical data.
- Blood, urine and vaginal swabs.

#### Cohort 2: unselected pregnant women (Fig. 6)

We are aiming for approximately 10,000 women to be recruited to this cohort across the three countries. These women will be approached when visiting the clinic for antenatal care and will be asked to provide data and biological samples to derive normative ranges for pregnant women in less-developed countries (PRECISE Objective 2b). We will recruit women age 14*- 49 years-old (*as pregnant adolescents are at increased risk of placental disease) [40]. For recruitment of women aged 14 - 17 years old we will be guided by the consent and assent processes advised by in-country ethics boards. Research partners in the study countries have advised that pregnant women aged 16 years and over will not require parental consent to participate but can provide consent themselves.

**Fig. 6:**
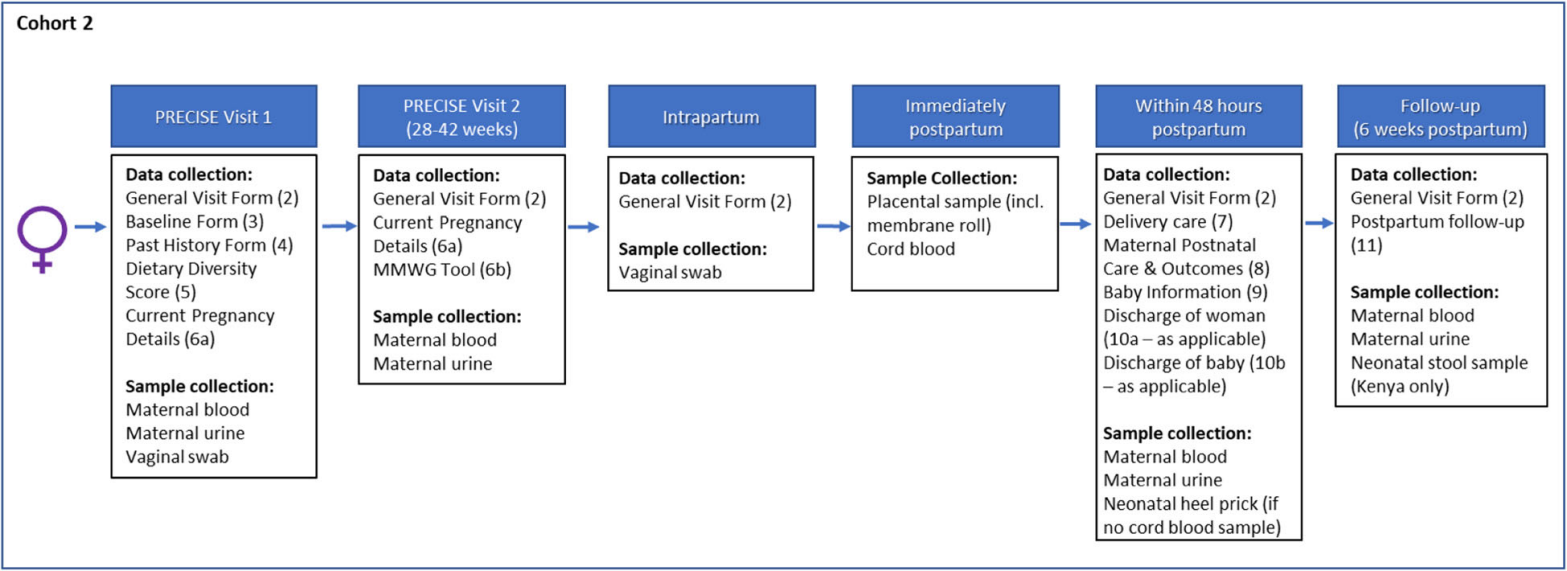
Flow and sampling of unselected pregnant women

#### Cohort 2: inclusion criteria

- Pregnant and attending facility for their own care or for the care of someone else.
- Have not been referred from another facility.

#### Cohort 2: exclusion criteria

- Does not plan to deliver at this facility.

#### Study recruitment (first study visit)

It is anticipated that the median gestational age at booking in each country will be about 24–27 weeks of pregnancy (unpublished data from the Community-Level Interventions for Pre-eclampsia [CLIP] trials).

Whenever possible, at the booking visit women will be approached for participation. Consenting women will have their pregnancies dated using, in order of accuracy, ultrasound dating (recognised to be very uncommon in these settings), certain last menstrual period, estimated last menstrual period and symphysis-fundal height (supplemented, for study purposes, by the ultrasound-based transcerebellar diameter (TraCer) device that determines gestational age using the highly-conserved transcerebellar diameter [41]); the TraCer dating will be used as the gold standard for the academic enterprise. As the TraCer device does not yet have a CE mark it is not appropriate that these data be used to direct clinical care; however, if during the scan there are concerns about the fetus or pregnancy, the women will be referred down the standard clinical care pathway for further investigation. If TraCer is CE marked during the clinical phase of PRECISE, these data will then begin being shared with clinical teams. Thereafter, the women will be asked to provide:

- Baseline demographic, non-clinical and clinical data.
- Blood, urine and vaginal swab.

Should a woman not be identified and recruited at her booking visit (and not declined participation), she will be approached at her next visit and, if informed consent is obtained, she will be recruited into PRECISE and the above-described data and samples obtained.

#### Third trimester

Between 28 weeks’ gestation and the onset of labour, and at least 4 weeks after the study recruitment visit, participating pregnant women will be asked to provide:

- Non-clinical and clinical data, including administration of the WHO maternal morbidity working group ANC tool [42].
- Blood and urine.

***Intrapartum***, the following will be collected:

- An intrapartum vaginal swab will be taken in association with a routine, clinically-indicated vaginal examination.

#### Immediately postpartum

Participating women will be asked to provide:

- Non-clinical and clinical data.
- Placental samples and cord blood.

#### Within 48 h postpartum

Prior to discharge home, and up to 48 h postpartum, participating women will be asked to provide:

- Non-clinical and clinical data.
- Blood and urine.

In addition, permission will be requested to gather the following neonatal data and samples:

- Clinical data.
- Neonatal heel prick (if no cord blood sample collected).

#### Six weeks postpartum

At, or soon as possible after, 6 weeks postpartum, participating women will be asked to provide:

- Non-clinical and clinical data.
- Blood and urine.

In addition, permission will be requested to gather the following neonatal data and samples:

- Non-clinical and clinical data.
- Neonatal stool sample (Kenya only)

#### Cohort 3 & 4: pregnant women at time-of-disease (Fig. 7)

Cohort 3 will include women who have pregnancies complicated by pregnancy hypertension (systolic blood pressure ≥ 140 mmHg or diastolic blood pressure ≥ 90 mmHg) or FGR (as suspected by the clinician undertaking their care). Cohort 4 includes women with who are either experiencing an intrauterine fetal death or who have delivered a stillborn infant. These women will be approached when visiting the clinic for antenatal care or if they have been referred for care due to suspected or diagnosed placental complications. We will recruit women age 14*-49 (*as pregnant adolescents are at high risk of placental disease). Research partners in the study countries have advised that pregnant women aged 16 and over will not require parental consent to participate but can provide consent themselves.

**Fig. 7:**
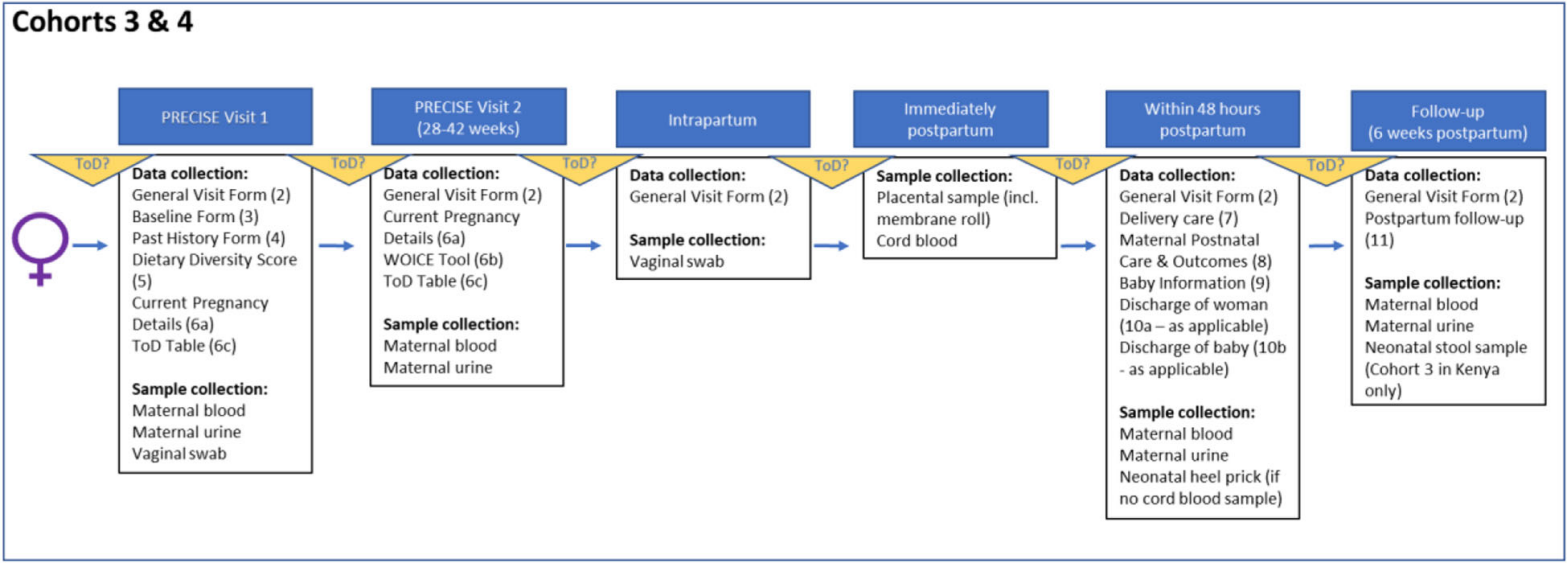
Flow and sampling of pregnant women with suspected/confirmed placental disease

**Fig. 8:**
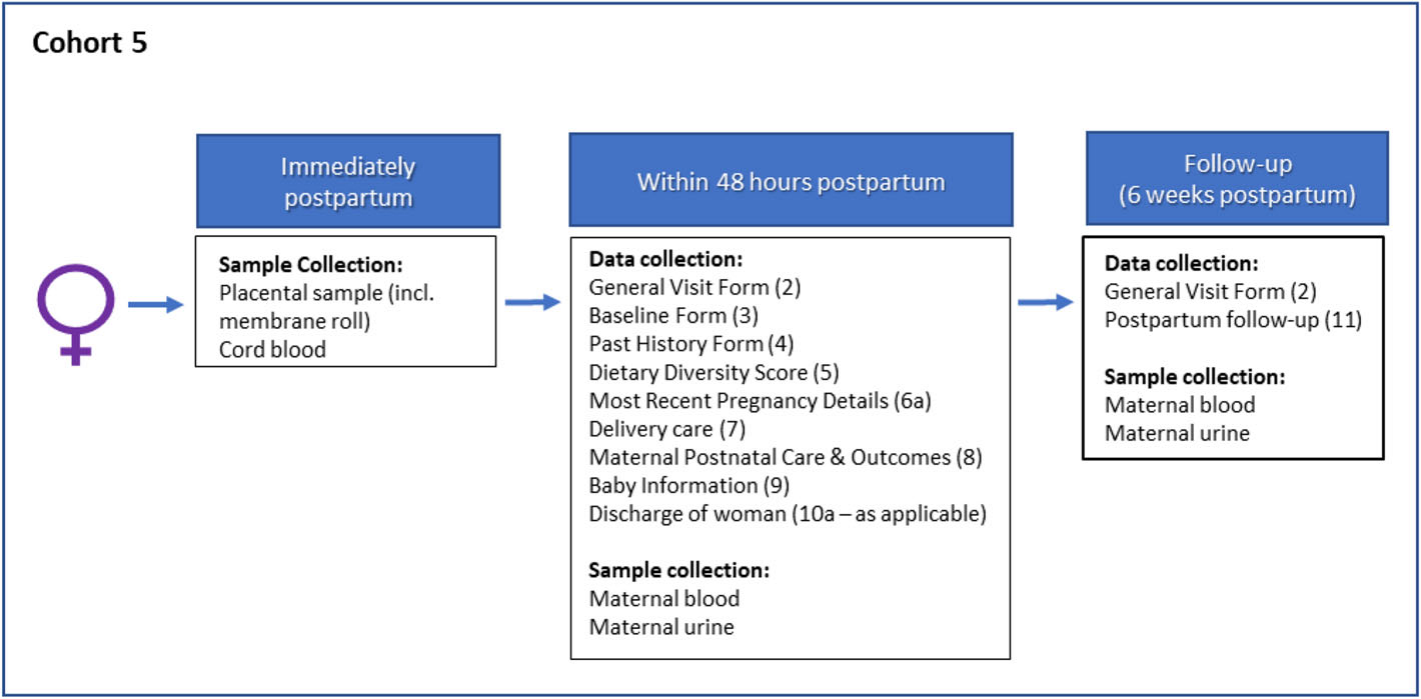
Flow and sampling of women with stillbirths recruited postpartum

#### Cohort 3: inclusion criteria

- Pregnant and attending facility for their own care.
- Can have been referred from another facility.
- By clinical assessment, have hypertension or suspected FGR (without hypertension)

#### Cohort 4: inclusion criteria

- Pregnant and attending facility for their own care.
- Can have been referred from another facility.
- By clinical assessment, have intrauterine fetal death (IUFD) or stillbirth.

#### Time-of-disease – cohorts 3 & 4

With the first diagnosis of pregnancy hypertension ≥20^+ 0^ weeks, clinical suspicion of fetal growth restriction, or fetal death in utero, women will be approached for recruitment and consent. Health workers will be trained in appropriate and sensitive recruitment of women in the time-of-disease cohort, acknowledging the emotional distress that women may be feeling at this time. Consenting pregnant women will be asked to provide:

- Non-clinical and clinical data.
- Blood, urine and vaginal swab.

#### Third trimester

Between 28 weeks’ gestation and the onset of labour, and at least four weeks after the study recruitment visit, participating pregnant women will be asked to provide:

- Non-clinical and clinical data.
- Blood and urine.

***Intrapartum***, the following will be collected:

- An intrapartum vaginal swab will be taken in association with a routine, clinically-indicated vaginal examination.

#### Immediately postpartum

Participating women will be asked to provide:

- Non-clinical and clinical data.
- A placental sample and cord blood.

#### Cohort 3 only - within 48 h postpartum

Prior to discharge home, and up to 48 h postpartum, participating women will be asked to provide:

- Non-clinical and clinical data.
- Blood and urine.

In addition, permission will be requested to gather the following neonatal data and samples:

- Clinical data.
- Neonatal heel prick (if no cord blood sample collected).

#### Cohort 4 only - within 48 h postpartum

Prior to discharge home, and up to 48 h postpartum, participating women will be asked to provide:

- Non-clinical and clinical data.
- Blood and urine.

In addition, permission will be requested to gather the following fetal/neonatal data:

- Clinical data.

#### Cohort 3 only - six weeks postpartum

At, or soon as possible after, 6 weeks postpartum, participating women will be asked to provide:

- Non-clinical and clinical data.
- Blood and urine.

In addition, permission will be requested to gather the following neonatal data and samples:

- Non-clinical and clinical data.
- Neonatal stool sample (Kenya only)

#### Cohort 4 only –six weeks postpartum

At, or soon as possible after, 6 weeks postpartum, participating women will be asked to provide:

- Non-clinical and clinical data.
- Blood and urine.

#### Cohort 5: women with stillbirths recruited postpartum (Fig. 8) (Kenya only)

For women who have been referred to the facility with IUFD and for whom it will not be possible or appropriate to approach and consent before delivery, consent to collect retrospective data and samples will be sought postpartum. We will recruit women age 14*-49 (*as pregnant adolescents are at high risk of placental disease) in the delivery suites of the PRECISE facilities. As for Cohorts 3 & 4, for recruitment of women aged 14 - 17 years old we will be guided by the consent and assent processes advised by in-country ethics boards. Research partners in the study countries have advised that pregnant women aged 16 and over will not require parental consent to participate but can provide consent themselves.

#### Cohort 5: inclusion criteria

- Referred to facility with IUFD or recently delivered a stillborn baby.

#### Immediately postpartum

Participating women will be asked to provide:

- A placental sample and cord blood.

#### Within 48 h postpartum

Prior to discharge home, and up to 48 h postpartum, participating women will be asked to provide:

- Non-clinical and clinical data.
- Blood and urine.

#### Six weeks postpartum

At, or soon as possible after, 6 weeks postpartum, participating women will be asked to provide:

- Non-clinical and clinical data.
- Blood and urine.

Women are not offered incentives to attend the follow up visit, however, in The Gambia and Mozambique, if needed, transport will be arranged to bring the woman and her baby to the unit but no money is given.

#### Collection of samples (Tables 2, 3, and 4)

##### Blood

Plasma samples will be used for placental growth factor and nutritional status assessments. All samples will be stored for later use in discovery science using semi-agnostic proteomics, genomics, epigenomics and metagenomics.

**Table 2:** Sampling from Cohort 1 (non-pregnant women of reproductive age)

Samples	Enrolment (Non-Pregnant)	Booking (Pregnant)	Antenatal (28^+ 1^–36^+ 6^)	Intra-partum	Immediately post-partum	Within 48 h postpartum	Total Collections
Blood	16 mL						1
Urine	20 mL						1
Vaginal swabs	4 swabs						1

**Table 3:** Sampling from Cohort 2 (unselected pregnant women) and Cohorts 3 & 4 (women at time-of-disease) and their infants

Samples	Booking	Antenatal	Intra-partum	Immediately postpartum	Within 48 h postpartum	6 weeks postpartum	Total Collections
Blood	16 mL	16 mL			16 mL	16 mL	4
Urine	20 mL	20 mL			20 mL	20 mL	4
Vaginal swabs	4 swabs		4 swabs				2
Cord blood				16 mL			1
Placental tissue				14 small pieces of tissue, membrane and cord			1
Newborn blood					2–3 drops from heel stick ONLY if cord blood is not collected		1
Neonatal stool						Approximately 100 mg	1^a^

^a^Cohorts 2 and 3 in Kenya only

**Table 4:** Sampling from Cohort 5 (women with stillbirth recruited postpartum)

Samples	Immediately postpartum	Within 48 h postpartum	6 weeks postpartum	Total collections
Blood		16 mL	16 mL	2
Urine		20 mL	20 mL	2
Cord blood	16 mL			1
Placental tissue	14 small pieces of tissue, membrane and cord			1

***Cohort 1:*** We anticipate that each participant enrolled in the non-pregnant cohort will provide one blood sample to the PRECISE Study at the time of enrolment. The blood draw will be approximately 16 mL.

***Cohort 2*** We anticipate that each participant enrolled in the pregnancy cohort will provide at least four blood samples to the PRECISE Study: one at booking; one later in pregnancy between 28^+ 1^–36^+ 6^ weeks’ gestation (at least 4 weeks after the initial biological sampling); one within 48 h postpartum and one at or after 6 weeks postpartum. Each blood draw will be approximately 16 mL (roughly equivalent to 1 tablespoon).

***Cohorts 3 & 4*** We anticipate that each participant enrolled in the pregnancy at ‘time-of-disease’ cohort will provide from one to four blood samples to the PRECISE study, depending upon when they present with complications: one at enrolment; and/or one later in pregnancy between 28^+ 1^–36^+ 6^ weeks gestation (at least 4 weeks after the initial biological sampling); one within 48 h postpartum and one at or after 6 weeks postpartum. Each blood draw will be approximately 16 mL (roughly equivalent to 1 tablespoon).

All blood collection is intended to qualify as minimal risk. The total volume and frequency, when considered in the context of the clinical encounter, will not exceed the following parameters (OHRP (45 CFR 46.110):

- For healthy, non-pregnant adults who weigh at least 50 kg, the amounts drawn may not exceed 550 mL in an 8-week period and collection may not occur more frequently than 2 times per week.
- For other adults and children, the amount drawn may not exceed the lesser of 50 mL or 3 mL per kg in an 8-week period and collection may not occur more frequently than 2 times per week.

***Cohort 5:*** We anticipate that participants enrolled in the stillbirth cohort who are recruited postpartum will provide two blood samples to the PRECISE Study: one at enrolment and one at or after 6 weeks postpartum. Each blood draw will be approximately 16 mL (roughly equivalent to 1 tablespoon).

##### Urine

Up to 20 mL of urine will be collected up to four times, at the same time the blood sample is collected for Cohorts 2, 3 and 4. For Cohort 1 (the Non-Pregnancy Cohort), one urine sample will be collected once at the time of enrolment. All samples will be stored for later use in discovery science using semi-agnostic proteomics and metagenomics.

##### Vaginal swabs

With explicit additional consent, mid-vaginal swabs will be collected at the booking visit and when the women present to deliver. For Cohort 1 (the Non-Pregnancy Cohort), vaginal swabs will be collected once at the time of enrolment. For cohort 5, we will not collect vaginal swabs. All samples will be stored for later use, primarily related to metagenomics to gain insights into the interaction between the vaginal microbiome and pregnancy, newborn and puerperal outcomes.

##### Cord blood

After delivery of the baby, the umbilical cord is clamped and cut. The umbilical cord (including the cord blood within it) is generally discarded along with the placenta. After the cord is clamped and cut, the cord blood will be collected. The amount of cord blood drawn will be approximately 16 mL (roughly equivalent to 1 tablespoon). All samples will be stored for later use in discovery science using semi-agnostic proteomics, genomics and epigenomics.

##### Placenta

The placenta will be trimmed and weighed and photographed using a digital camera in a fixed position. Eight small coin-sized tissue samples will be taken from the placenta, five 5 mm-long sections taken from the cord and one strip of membrane. Four samples will be processed in formalin for histology and the rest will be flash frozen in liquid nitrogen for proteomic and metabolomic analyses. All samples will be stored for later use in discovery science correlating placental findings with clinical outcomes and the outputs from the ‘omics science platforms.

##### Newborn blood

Only if the cord blood collection is missed, 2–3 drops of blood for DNA will be collected from a heel stick prior to discharge from the facility. All samples will be stored for later use in discovery science using semi-agnostic proteomics, genomics and epigenomics.

##### Neonatal stool (Kenya only)

The stool will be collected to examine the newborn microbiome. The neonatal stool sample will be collected from the baby’s nappy at the 6 weeks postpartum follow-up visit.

The samples will be collected, processed and stored in adherence to the PRECISE Network biorepository standard operating procedures (SOPs) across all sites. Study personnel at each site will receive initial and ongoing training as needed to ensure SOPs are followed and samples are of the highest quality.

### Interactions with the clinical service

As PRECISE will improve the quality of antenatal care provision, with accurate blood pressure measurement, a focus on stillbirth, and routine pulse oximetry, the research team recognises that defined clinical responses are incumbent on us, primarily through referral into existing clinical pathways. We are determining if and how to add mental health elements to the protocol through qualitative research. An important piece of prior work is to identify the clinical pathways for women identified with mental health emergencies.

### Co-ordination

Academic co-ordination and general oversight are shared between the central PRECISE teams located at KCL and UBC. In-country clinical co-ordination is supplied by the research teams located in Farafenni (The Gambia), Mombasa (Kenya) and Manhiça (Mozambique), with central support in Fajara and Nairobi, in The Gambia and Kenya, respectively. Details of the monitoring and quality assurance plans are included in the database and biorepository papers (Craik R, et al.: Baobab Laboratory Information Management System, submitted).

### Statistical approach

Our two principal analytical approaches will be to identify factors that predict the onset of placental disorders and factors that identify women, fetuses and newborns at incrementally increased risk at time-of-disease with pregnancy hypertension and FGR. We will prioritise modifiable factors to accelerate clinical improvements. The detailed statistical plan is being developed and will be published in due course.

## Discussion

This protocol paper summarises the clinical aspects of the PRECISE Network’s field activities and, as stated, should be read alongside the database and biobanking papers for a full understanding of the scope and detail of what is now underway in The Gambia, Kenya, and Mozambique.

To accelerate progress towards the women’s and children’s health-relevant Sustainable Development Goals, we need to understand how a variety of social, chronic disease, biomarker and pregnancy-specific determinants health interact to result in either a resilient or a compromised pregnancy for either mother or fetus/newborn, or both. It is our opinion that this protocol has been designed to create such a depth of understanding.

We are seeking funding to maintain the cohort to better understand the implications of pregnancy complications for both maternal and child health, as well as additional funds to begin intervention studies (e.g., stepped-wedge cluster randomised controlled trials) to convert what we already know and what has been learnt from PRECISE into clinical care in the communities that have dedicated themselves to a shared PRECISE vision and agenda.

Our findings will be of interest to the women and their communities, the local and national health systems, scientific colleagues, health advocates and funders. We have developed a comprehensive and multi-layered communications strategy to reach out to each of these groups, whether through direct oral interactions, infographics, presentations at scientific (e.g., Society for Reproductive Investigation) or advocacy (e.g., Women Deliver) meetings, policy briefs and scientific papers. By embedding our advocacy and engagement within governments, multilaterals and NGOs, we envision local implementation of interventions demonstrated through the PRECISE study to be robust and effective reducing maternal and neonatal mortality and morbidity.

## Conclusion

The name for PRECISE implies a commitment to precision medicine, and this protocol has the potential to lead to the provision of individualised, precision health care to women and their infants whether they encounter the health system on their doorsteps or at the tertiary referral hospital.

## Data Availability

Not applicable to this manuscript (please database and biobanking papers for open-access policies).

## Abbreviations

AKU: Aga Khan University
ANC: Antenatal clinic
CHV: Community health volunteers
CISM: Centro de Investigação de Saúde de Manhiça
CLIP: Community-Level Interventions for Pre-eclampsia
FGR: Fetal growth restriction
IUFD: Intrauterine fetal death
PHC: Primary health centre
PIERS: Pre-eclampsia Integrated Estimate of RiSk
POM: PIERS On the Move
SOP: Standard operating procedures
UKRI: UK Research and Innovation
